# Determinants of Receiving the Pandemic (H1N1) 2009 Vaccine and Intention to Receive the Seasonal Influenza Vaccine in Taiwan

**DOI:** 10.1371/journal.pone.0101083

**Published:** 2014-06-27

**Authors:** Ta-Chien Chan, Yang-chih Fu, Da-Wei Wang, Jen-Hsiang Chuang

**Affiliations:** 1 Research Center for Humanities and Social Sciences, Academia Sinica, Taipei, Taiwan, Republic of China; 2 Institute of Sociology, Academia Sinica, Taipei, Taiwan, Republic of China; 3 Institute of Information Science, Academia Sinica, Taipei, Taiwan, Republic of China; 4 Deputy Director-General’s Office, Centers for Disease Control, Taipei, Taiwan, Republic of China; 5 Institute of Public Health, National Yang-Ming University, Taipei, Taiwan, Republic of China; The University of Adelaide, Australia

## Abstract

**Objectives:**

The paper examines the factors associated with both receiving pandemic (H1N1) 2009 vaccines and individuals’ intentions to get the next seasonal influenza vaccine in Taiwan.

**Methods:**

We conducted a representative nationwide survey with in-person household interviews during April–July 2010. Multivariate logistic regression incorporated socio-demographic background, household characteristics, health status, behaviors, and perceptions of influenza and vaccination.

**Results:**

We completed interviews with 1,954 respondents. Among those, 548 (28.0%) received the pandemic (H1N1) 2009 vaccination, and 469 (24.0%) intended to get the next seasonal influenza vaccine. Receipt of the H1N1 vaccine was more prevalent among schoolchildren, the elderly, those who had contact with more people in their daily lives, and those who had received influenza vaccinations in previous years. In comparison, the intention to receive the next seasonal influenza vaccine tended to be stronger among children, the elderly, and those who reported less healthy status or lived with children, who received a seasonal influenza vaccination before, and who worried more about a possible new pandemic.

**Conclusions:**

Children, the elderly, and those who had gotten seasonal flu shots before in Taiwan were more likely to both receive a pandemic H1N1 vaccination and intend to receive a seasonal influenza vaccine.

## Introduction

Epidemic and pandemic influenza infections cause tremendous social impacts in addition to generating serious threats to the health and lives of the global population [1], [2]. To reduce such threats and impacts, governments have implemented or promoted two primary public-health approaches to prevent and control influenza: a pharmaceutical approach such as vaccinations [3] and anti-viral drugs [4], and a non-pharmaceutical approach such as self-protective behavior [5] and social distancing [6]. As is the case with many other diseases, prevention always yields better outcomes than controlling the epidemic afterwards. Among current prevention policies, influenza vaccinations for children and the elderly have proven to be a highly cost-effective method to prevent people from getting severe influenza illnesses [7], [8]. Other high-risk subpopulations, such as healthcare workers, poultry workers, and people with chronic diseases, are also among the priority groups to receive influenza vaccines.

Due to the limited budget for purchasing influenza vaccines and for other reasons, vaccination coverage rates vary widely across countries. According to surveys in 11 European countries during the 2007/08 influenza season [9], for example, coverage rates ranged from 9.5% to 28.7% among the general population, from 13.9% to 70.2% among the elderly, and from 4.2% to 19.3% among children. In the non-western countries that have implemented various policies related to prevention and control of influenza, the vaccine coverage rate has been a key issue, while policy-makers strive to keep a reasonable balance between effective coverage and cost-effectiveness.

In Taiwan, government-funded influenza vaccinations started in 1998, when the coverage rate among the elderly was only 9.9% [10]. As in many other countries, Taiwan’s annual budget for purchasing the influenza vaccine is too limited to cover the whole population. The influenza vaccination policy in Taiwan has gradually expanded from high-risk populations to key spreaders to further reduce the number of cases and deaths. Therefore, the government announces the priority groups for vaccinations every year based on recommendations from the Advisory Committee on Immunization Practices (ACIP).

High-risk subpopulations have a chance to receive vaccinations free of charge beginning in October of each year. Because such policies create significant incentives to receive vaccines, we summarize the seasonal and pandemic vaccination plans during the 2009/2010 influenza season (Table 1). The priority groups and starting dates of vaccination differed between the (H1N1) 2009 pandemic and seasonal influenza. In particular, the priority groups for receiving pandemic (H1N1) 2009 vaccines covered more subpopulations than those for receiving seasonal influenza ones, such as the residents affected by typhoon Morakot, infants, pregnant women, schoolchildren between grades 5 and 12, and adults ages 19–24.

**Table 1:** Priorities for the influenza vaccination plan in Taiwan during the 2009–2010 influenza season.

Pandemic (H1N1) 2009 vaccine		Seasonal influenza vaccine	
Priority groups	Starting Date	Priority groups	Starting Date
Residents affected by Typhoon Morakot	Nov. 1	Adults ages ≥65 years of age	Oct. 1
Healthcare and public health workers	Nov. 2	Patients and workers at long-term care centers	Oct. 1
Infants ages 6–11 months	Nov. 9	Grade 1–4 schoolchildren with school-based vaccination	Oct. 1
Pregnant women	Nov. 16	Children ages 6 months - 6 years	Oct. 1
Children ages 1–6 years	Nov. 16	People with severe illnesses/injuries	Oct. 1
People with severe illnesses/injuries	Nov. 16	Healthcare and public health workers	Oct. 1
Elementary school students with school-based vaccination	Nov. 16	Poultry and livestock industry workers	Oct. 1
Middle school students with school-based vaccination	Nov. 23	People engaged in animal disease control	Oct. 1
High school students with school-based vaccination	Nov. 30	All others	Dec. 1
Adults ages 19–24 years	Dec. 1		
People with specific chronic diseases	Dec. 7		
All others	Dec. 12		

The seasonal influenza vaccination was launched earlier, on October 1, 2009, and the pandemic (H1N1) 2009 vaccination started on November 1, 2009, followed by a few waves of vaccinations for different priority groups. During the 2009/10 influenza season, 11.9% of the population received at least one dose of the seasonal influenza vaccine, including 28.5% of children ages 6 months to 6 years, 79.6% of schoolchildren in grades 1 to 4, and 37.6% of the elderly, according to the Influenza Vaccine Information System (IVIS) [11]. In contrast, 24.6% of the population received at least one dose of the pandemic (H1N1) 2009 vaccine, including 36.3% of persons ages 6 months to 6 years, 75.2% of persons ages 7–18 years, and 13.9% of persons ages ≥19 years by December 1, 2010, according to the IVIS (unpublished data).

Despite the advancement of public health and increased budget allocation, the current level of vaccination coverage in Taiwan, as well as in Europe and the United States, was unable to reach the effectiveness of herd immunity [12]. Therefore, examining the reasons why members of the public receive or do not receive the influenza vaccination helps provide crucial information for promoting future vaccination campaigns and enhancing preparedness for the next pandemic.

One such reason lies in people’s motivation or intention. Most studies have shown that past experience with seasonal influenza vaccination is an important factor for understanding the intention to be vaccinated during the next influenza season. Like past experiences, the intention to be vaccinated is also likely to lead to actually getting vaccinated against influenza. Such behaviors and intentions, in turn, have been attributed to various socioeconomic and psychosocial factors, including gender, education status, risk perception of influenza, and trust of influenza safety [13]–[16]. Other factors, including the number and composition of household members as well as interpersonal contact intensity, may also affect individuals’ decisions or intentions to be vaccinated. As found in recent studies, for example, vaccine histories of other household members play a role in distinguishing who is more inclined to get vaccinated. One’s social networks have also been associated with decisions to get vaccinated, including parents’ decisions [17], [18]. Furthermore, those who have more contacts in their everyday lives, such as children and teenagers, may become victims or spreaders of the disease more easily [19]. Therefore, these factors should be incorporated into modeling why people vary in both behaviors and intentions to receive vaccinations.

In line with the well-recognized linkage between past and future vaccination uptakes, we contribute to the study of influenza vaccination in two ways. First, we identify and compare what kinds of socioeconomic, household, and psychosocial factors are associated with both the behavior (H1N1 2009 pandemic) and intention of vaccination uptakes. Second, we incorporate both the experience and perception of a pandemic (H1N1 2009) influenza into modeling the intention to receive the vaccination during the next seasonal influenza.

With a systematic analysis of data collected from a nationwide representative survey in 2010, we provide insights into the cognitive differences between the actual vaccination behavior (such as past seasonal influenza vaccination history and pandemic (H1N1) 2009 influenza vaccination) and the intention to get vaccinated. Such insights should yield policy implications for influenza vaccination strategy and risk management during the next pandemic.

## Materials and Methods

### Ethics

This study was approved by the institutional review board (IRB) of the Academia Sinica for research ethics (IRB#: AS-IRB-HS 02-13020). To minimize potential ethical issues that might arise during data collection, we took several steps to protect our research subjects. First, all field interviewers and research staff signed an agreement on maintaining the confidentiality of respondents’ personal information. Second, when respondents were younger than age 8, we asked a parent or a guardian to answer the questions on the child’s behalf. Third, we required written informed consent prior to the interviews from respondents, including parents or guardians if the respondents were under 18 years old. Fourth, we maintained all individual survey results under anonymity and retained none of the identifiable personal information (including the names listed in the contact diaries). Finally, after data collection, we destroyed the sample list; and after data cleaning, we destroyed the completed paper questionnaires [20].

### Survey Design

Following our extensive experiences with nationwide general social surveys in Taiwan, we designed a representative survey that included questions about residents’ knowledge of and experiences with both the pandemic (H1N1) 2009 and seasonal influenza, as well as 24-hour contact diaries among the whole population [20]. We conducted the survey with in-person household interviews. To understand how contact patterns were linked to the transmission of influenza, we added contact diaries that were slightly revised from those used in similar studies in Europe and Asia [21]–[23]. For each physical contact and face-to-face contact with verbal communication made within 2 meters (up to 40 persons), we asked respondents to record location, duration, initiation, and other features of the contact situations.

We ensured that our successful survey sample would be as representative as possible by adopting two critical survey designs, as detailed in a previous report [20]. First, we followed the 3-stage probability sampling scheme that the Taiwan Social Change Survey has used for decades [24]. To cover the total population, we sampled from all residents who were listed in the Household and Population Register in Taiwan. Second, during the field interview we allowed no substitute respondents.

### Asking about Vaccination: Behaviors and Intentions

We asked our respondents both “Have you received a pandemic (H1N1) 2009 vaccination?” and “Will you receive a seasonal influenza vaccination this coming flu season?” The answer to the pandemic (H1N1) 2009 vaccination was simply either Yes or No. For the seasonal influenza vaccination, we pooled the original answers into a dichotomy, indicating the intention to receive the vaccination (including “definitely will” and “probably will”) or not to receive the vaccination (including “probably will not,” “definitely will not,” and “don’t know”).

### Socio-demographic and household characteristics

We used such socio-demographic variables as gender, age groups, education status, and work status as the adjusted confounders in the models. The classifications of age groups differed in the two analyses because the vaccination policy assigned different priority groups for the pandemic (H1N1) 2009 and regular seasonal influenza in Taiwan. The survey did not track household members’ vaccination history. Nor did it ask these members about their intentions toward vaccination. As an alternative, we examined whether one’s cohabitants included someone who belonged to a high-risk subpopulation or was a medical professional. In the analysis, we examine whether respondents’ behaviors and intentions regarding influenza vaccination vary by the number of contacts, the percentage of bodily contacts, the number of household members, the numbers of children and the elderly, and whether someone in the household worked in the medical industry.

To compare high and low frequencies of the number of household members and contact number, and partly to simplify the analyses, we selected the median values as the thresholds. Variables used for the household included whether the number of household members was equal to or larger than 5 (median = 4), whether there was any household member with a medical background, and whether there was any household member under age 12 or over age 65. Furthermore, we constructed two measures from the contact diaries to indicate contact intensity: whether the number of contacts with people within the past 24 hours was equal to or larger than 10 (median = 9), and the percentage of bodily contacts among all contacts within 24 hours.

### Health status, behaviors, and perceptions

We also took into account several other factors that might affect the behaviors of receiving and intentions to receive influenza vaccinations. The self-reported health condition reflected how respondents perceived their health condition in general. We recoded the answering categories, from 1 to 5, into: poor, fair, good, very good, and excellent, so that a higher score always indicates a better health condition. How often one went to public places (from 1 “almost never” to 5 “almost every day”) serves as a proxy indicator of exposure to the influenza virus from human gatherings and environmental contamination. Watching intense political talk shows about the adverse effects of influenza vaccination may affect how one perceives the safety of vaccinations, which in turn may influence individuals’ intentions and subsequent behaviors. Both the perception of the severity of the (H1N1) 2009 pandemic in Taiwan and the level of worry about a new pandemic serve as measurements of risk perception. Respondents’ past experience of influenza vaccinations could also be an important factor: If respondents had received influenza vaccination at least once in the past five years, we coded their experience as 1, and those with no vaccination were coded as 0.

### Statistical analysis

We first used Pearson’s chi-square test to compare the categorical variables’ frequency distributions, and the Wilcoxon rank-sum test to compare the continuous variables’ medians in the two separate analyses, one about the pandemic (H1N1) 2009 vaccination, and the other about the intention to receive a seasonal influenza vaccination. Due to different age limits set for government-funded vaccination plans, we used age groups under age 18 in two ways (ages 0–10 and ages 0–18) when analyzing who received the pandemic H1N1 vaccination and who intended to get the seasonal influenza vaccine. For the pandemic (H1N1) 2009 vaccine, all children under age 18 belonged to the priority group for free vaccination (Table 1). For the seasonal influenza vaccine, only children under 10 (up to grade 4 schoolchildren) were on the priority list.

We selected the variables that had a p-value less than 0.2 for the stepwise multivariate logistic regression to determine the factors associated with receiving pandemic (H1N1) 2009 vaccines and the intention to get influenza shots in the next season and to estimate their adjusted odds ratios (AOR) with 95% confidence intervals (CI) as well. The selection criterion of p-value used for both the chi-square test and the Wilcoxon rank sum test served to filter out the variables unrelated to the dependent variables [25]. To assess the performance of the final multivariate model, we used the Hosmer-Lemeshow goodness-of-fit test and the area under the receiver operating characteristic (ROC) to measure calibration and discrimination, respectively. A two-sided p-value of less than 0.05 was considered statistically significant. All statistical analyses were performed with Stata (StataCorp LP, College Station, TX). The summarized list of the variables and their responses in this study are shown in Table S1.

## Results

The survey data of this study were collected during April–July 2010, immediately after the (H1N1) 2009 pandemic in Taiwan. We finished the survey with 1,954 cases of individual questionnaires and 1,943 24-hour contact diaries, at a response rate of 51%. There were 548 (28.0%) respondents having received pandemic (H1N1) 2009 vaccines and 469 (24.0%) persons classified as intending to get seasonal influenza vaccines in the next season, respectively.

### Factors on receiving a pandemic (H1N1) 2009 vaccination

The socio-demographic variables, such as age group, education, and working status, all turned out to be significant factors in distinguishing between those who received a pandemic (H1N1) 2009 vaccination and those who did not (all p<0.001, Table 2). The youngest age group (i.e., 0–18 years old) had the highest vaccination rate (65.7%; 318/484), because they were the priority groups, and all schoolchildren received school-based pandemic (H1N1) 2009 vaccination services. Respondents with better self-reported health condition, higher frequency of visiting public places, and higher risk perception of the 2009 pandemic and future pandemics were all more likely to receive a pandemic (H1N1) 2009 vaccination (details in Table S2). In contrast, respondents who had a habit of watching political talk shows were less likely to receive a pandemic (H1N1) 2009 vaccination. If respondents believed that the pandemic in 2009 was serious, or had the past experiences of getting seasonal influenza shots, they were also more likely to get the pandemic (H1N1) 2009 shot. In addition, higher percentages of receiving pandemic (H1N1) 2009 vaccinations prevailed among those who lived in a large household (with at least 5 members), who had contacts with more people (≧10 persons) during the past 24 hours, and who had more bodily contact with individuals out of these daily contacts.

**Table 2:** Selected variables associated with receipt of pandemic (H1N1) 2009 vaccination.

Variables	Did not receive Vaccination		Received Vaccination		*p*-value
	*n = 1,406*		*n = 548*		
Gender					0.691
Male, no.(%)	712	(50.6)	283	(51.6)	
Female, no.(%)	694	(49.4)	265	(48.4)	
Age groups, no. (%)					<0.001
0–18	166	(11.8)	318	(58.0)	
19–35	411	(29.2)	42	(7.7)	
36–50	359	(25.5)	54	(9.9)	
51–64	309	(22.0)	45	(8.2)	
65 & older	161	(11.5)	89	(16.2)	
Education, no. (%)					<0.001
Elementary	367	(26.2)	257	(46.9)	
High school	548	(39.1)	210	(38.3)	
College or higher	488	(34.8)	81	(14.8)	
Working status, no. (%)					<0.001
Pre-school	90	(6.4)	49	(8.9)	
Students	143	(10.2)	269	(49.1)	
Work	793	(56.4)	123	(22.5)	
Unemployed	379	(27.0)	107	(19.5)	
Self-reported health status, median (IQR)	3	(2–4)	3	(2–4)	<0.001
Frequency of visiting public place, median (IQR)	4	(3–5)	5	(4–5)	<0.001
Habit of watching political talk shows, no. (%)	497	(35.4)	112	(20.4)	<0.001
Perception of severity of pandemic in 2009, median (IQR)	3	(2–3)	3	(2–4)	<0.001
Level of worry about new pandemic, median (IQR)	3	(2–3)	3	(2–3)	0.041
Previous vaccination against seasonal influenza, no. (%)	231	(16.4)	320	(58.4)	<0.001
Household, no. (%)					
# members> = 5	554	(39.4)	257	(46.9)	0.003
with med. background	135	(9.6)	52	(9.5)	0.951
someone under age 12	449	(31.9)	191	(34.9)	0.217
someone over age 65	388	(27.6)	169	(30.8)	0.154
Contact diary					
# people> = 10, no. (%)	669	(47.9)	322	(59.1)	<0.000
% bodily contact, median (IQR)	0.24	(0.1–0.2)	0.38	(0.2–0.7)	<0.000

IQR interquartile range (25th percentile–75th percentile).

In the final model of the multivariate logistic regression (Table 3), higher percentages of children (ages 0–18, AOR: 23.5; 95% CI: 11.8–46.8) and the elderly (AOR: 2.2; 95% CI: 1.2–4.0) received pandemic (H1N1) 2009 vaccination than adults ages 19–35 years. When we examine respondents’ study or work statuses, pre-schoolers had the lowest probability of getting the pandemic (H1N1) 2009 vaccination (AOR: 0.2; 95% CI: 0.1–0.4). Respondents with past influenza vaccination experiences had more opportunities to get pandemic (H1N1) 2009 vaccinations (AOR: 6.4; 95% CI: 4.7–8.9). Respondents who had contact with more people (≧10 persons) in one day were more likely to prefer getting pandemic (H1N1) 2009 vaccines (AOR: 1.4; 95% CI: 1.0–1.8). The overall ROC of the model was 86.4%, and the results of the Hosmer-Lemeshow goodness-of-fit test showed that the data were well fitted by the model (p = 0.24).

**Table 3:** Multivariate logistic regression for covariates of receiving pandemic (H1N1) 2009 vaccination.

Variables	Multivariate Analysis		
	Odds Ratio	95% CI	*p*-value
Age groups			
0–18	23.5	11.8–46.8	<0.001
19–35	1 (Reference)		
36–50	1.5	0.9–2.3	0.128
51–64	1.3	0.8–2.2	0.331
65 & older	2.2	1.2–4.0	0.007
Working status			
Pre-school	0.2	0.1–0.4	<0.001
Students	1.3	0.6–2.8	0.457
Work	1.0	0.6–1.5	0.973
Unemployed	1 (Reference)		
Previous vaccination against seasonal influenza	6.4	4.7–8.9	<0.001
Contact diary			
# people> = 10	1.4	1.0–1.8	0.043

### Factors on the intention to receive seasonal influenza vaccines

Socio-demographic variables, such as age group, education, and working status, were all significant factors (p<0.001) affecting respondents’ decisions to receive seasonal influenza vaccines (Table 4). The major groups with no intention to get seasonal influenza vaccines were adults, those having a high-school educational level, and workers. Respondents who watch political talk shows preferred not to receive seasonal influenza vaccines. If respondents perceived higher levels of worry about future pandemics or had previous experiences in receiving the seasonal influenza vaccination, they were willing to get a seasonal influenza vaccine in the coming influenza season. Regarding household characteristics, those in larger households (household members≧5), households having members with a medical background, and those having members under age 12 or over 65 were more willing to receive seasonal influenza vaccines. Respondents with more bodily contacts in a single day were also more willing to receive seasonal influenza vaccines.

**Table 4:** Selected variables associated with intention to get seasonal influenza vaccines.

Variables	Do not intend to get vaccine		Intend to get vaccine		*p*-value
	*n = 1,485*		*n = 469*		
Gender, no.(%)					0.407
Male	764	(51.5)	231	(49.3)	
Female	721	(48.6)	238	(50.8)	
Age groups, no. (%)					<0.001
0–10	134	(9.0)	108	(23.0)	
11–18	180	(12.1)	62	(13.2)	
19–35	404	(27.2)	49	(10.5)	
36–50	353	(23.8)	60	(12.8)	
51–64	300	(20.2)	54	(11.5)	
65 & older	114	(7.7)	136	(29.0)	
Education, no. (%)					<0.001
Elementary	366	(24.7)	258	(55.0)	
High school	630	(42.5)	128	(27.3)	
College or higher	486	(32.8)	83	(17.7)	
Working status, no. (%)					<0.001
Pre-school	78	(5.3)	61	(13.0)	
Students	298	(20.1)	114	(24.3)	
Work	766	(51.6)	150	(32.0)	
Unemployed	342	(23.1)	144	(30.7)	
Self-reported health status, median (IQR)	3	(2–4)	2	(2–4)	0.055
Frequency of visiting public place, median (IQR)	4	(3–5)	4	(3–5)	0.964
Habit of watching political talk shows, no. (%)	485	(32.7)	124	(26.4)	0.011
Perception of severity of pandemic in 2009, median (IQR)	3	(2–3)	3	(2–3)	0.436
Level of worry about new pandemic, median (IQR)	3	(2–3)	3	(2–3)	0.005
Previous vaccination against seasonal influenza, no. (%)	194	(13.1)	357	(76.1)	<0.001
Household, no. (%)					
# members> = 5	593	(39.9)	218	(46.5)	0.012
with med. background	130	(8.8)	57	(12.2)	0.029
someone under age 12	460	(31.0)	180	(38.4)	0.003
someone over age 65	395	(26.6)	162	(34.5)	0.001
Contact diary					
# people> = 10, no. (%)	742	(50.3)	249	(53.3)	0.251
% bodily contact, median (IQR)	0.25	(0.1–0.8)	0.33	(0.1–0.7)	<0.001

IQR interquartile range (25th percentile–75th percentile).

In the final model of the multivariate logistic regression (Table 5), children (ages 0–10 years, AOR: 2.4; 95% CI: 1.5–4.0) and the elderly (AOR: 3.0; 95% CI: 1.8–4.9) were more willing to get seasonal influenza vaccines than adults ages 19–35 years. Respondents who reported being healthier were less likely to get seasonal influenza vaccines (AOR: 0.9; 95% CI: 0.8–1.0). Those who worried the most about a new pandemic (AOR: 1.3; 95% CI: 1.1–1.5) and who had previously received a seasonal influenza vaccination (AOR: 16.1; 95% CI: 11.9–21.5) were more willing to get the next seasonal influenza vaccine. If someone in the household was under age 12, respondents were more likely to receive seasonal influenza vaccines (AOR: 1.4; 95% CI: 1.0–1.9). The overall ROC of the model was 85.1%, and the results of the Hosmer-Lemeshow goodness-of-fit test showed that the data were well fitted by the model (p = 0.81).

**Table 5:** Multivariate logistic regression for covariates of intention to get seasonal influenza vaccines.

Variables	Multivariate Analysis		
	Odds Ratio	95% CI	*p*-value
Age groups			
0–10	2.4	1.5–4.0	0.001
11–18	1.4	0.8–2.3	0.208
19–35	1 (Reference)		
36–50	1.3	0.8–2.0	0.323
51–64	1.3	0.8–2.0	0.403
65 & older	3.0	1.8–4.9	<0.001
Self-reported health status	0.9	0.8–1.0	0.050
Level of worry about new pandemic	1.3	1.1–1.5	0.003
Previous vaccination against seasonal influenza	16.1	11.9–21.5	<0.001
Household			
someone under age 12	1.4	1.0–1.9	0.030

## Discussion

In this study, we have explored the factors that influenced whether the general public received pandemic (H1N1) 2009 vaccinations and, at the same time, their intentions to get seasonal influenza vaccines in the coming influenza season. Most current surveys have separated the actual vaccination factors and intentions into different surveys [26], [27], and thus the possibility of making comparisons between them is limited or unlikely. Furthermore, the percentage of those getting pandemic (H1N1) 2009 vaccines (28.0%) in this study was very close to the coverage rate (24.6%) reported by the IVIS. Although pandemic (H1N1) 2009 vaccinations covered a greater portion of the population than seasonal influenza vaccinations in Taiwan, our study showed that previous influenza vaccination experience [13], [15], [28], priority groups with government-funded vaccines (such as children and the elderly), and school-based vaccination programs played crucial roles in both receiving pandemic vaccinations and intention to receive vaccines.

One study on pandemic (H1N1) 2009 vaccination intention in Taiwan [16] showed that those who intended to get pandemic (H1N1) 2009 vaccines tended to be males, young adults (ages 18–24 years), the elderly, and those living in households with a mid-range monthly income (USD$ 1,667–3,333). It showed that 75.3% of the respondents ages 18–24 years, who were mostly college students, intended to get the pandemic (H1N1) vaccination. In comparison, respondents ages 25–44 had the weakest intentions to get the pandemic (H1N1) vaccination. According to the vaccination plan (the left column of Table 1), adults ages 19–24 years were one of the targeted groups **(**albeit among the last three**)** of government-funded pandemic (H1N1) vaccinations. Thus, the high intention within this group might be due to the promotion of free vaccinations.

The actual pandemic (H1N1) vaccination rate in the 19–35 age group, however, was only 9.27% (42 out of 453) in our study, which was the lowest vaccination rate among all age groups. The fact that few young adults got pandemic (H1N1) shots might be due to the passing of the pandemic peak (Nov. 22–Nov. 28, 2009) before they were eligible to get immunized [29]. A similarly low influenza vaccination rate among college students was also found in Hong Kong [30].

The previous study in Taiwan [16] also showed that those who intended to receive pandemic (H1N1) 2009 vaccines were more likely to perceive the pandemic as being more severe, to believe the H1N1 vaccine is more effective, and to foresee a lower barrier to receiving vaccinations in general. Unlike this previous study, gender was not a significant factor for receiving pandemic (H1N1) 2009 vaccines according to our findings. The elderly group and respondents who perceived higher pandemic risk, however, also had higher intention to receive seasonal influenza vaccines, which is similar to findings in Huang et al. for Taiwan [16] and Liao et al. for Hong Kong [31]. In other words, if respondents perceived their health status as good, they had lower intention to receive a seasonal influenza vaccine.

Compared with schoolchildren, pre-school children (ages ≦6 years) were found to have a lower pandemic (H1N1) vaccination rate. The possible reasons might be accessibility and concerns about vaccine safety. During the pandemic period, the vaccination campaign was implemented in all school settings (including elementary, middle, and high schools), which made the campaign more efficient and extensive. It was hard to reach pre-school children without parents’ help, however. The other potential concern was the safety of the pandemic influenza vaccines for young children. One review study in Europe found that the vaccine effectiveness was moderate to good, and that the safety of non-adjuvanted trivalent inactivated influenza vaccines was excellent among children [32]. In addition, the vaccination among children was also a cost-effective approach for preventing influenza disease burden [33]. How to enhance pre-school children’s vaccination coverage rate will be an ongoing challenge for public-health workers.

There were some concerns related to the validity of the responses from the children under age 8. In our survey, we instructed the interviewers that “if the targeted respondents were under age 8, all interviews and records must be answered and taken by a parent or a guardian.” So the information obtained under such circumstances refer to that received from the parent. In addition, because Taiwan’s civil law stipulates that children under age 8 lack behavioral competence, the acceptance of vaccination must be decided by a parent or guardian.

We included all age groups in the analysis, because the priority groups for free influenza vaccinations were set mostly by age limits, and young children are often a top priority. To verify that the results were not altered by the inclusion of young children, we conducted a separate analysis on the respondents under age 8. There were 168 respondents in this subsample (i.e., ages 0–7, about 8.6% of the full sample). The results from the analysis of this subsample in terms of both receiving the pandemic (H1N1) 2009 vaccination and intention to get the seasonal influenza vaccine showed that only receipt of the previous seasonal influenza vaccination was a significant explanatory factor (for receiving the pandemic 2009 vaccination, odds ratio = 4.01, 95% CI: 1.8–9.2, p<.001; for intention to take the next seasonal influenza vaccine, odds ratio = 8.75, 95% CI: 3.6–21.5, p<.001). Because such results are identical to those from the full sample, the inclusion of young children under age 8 in the models did not distort the analysis.

From the viewpoint of vaccination policy, it was desirable to examine why some respondents received either the pandemic (H1N1) 2009 vaccine or past seasonal influenza vaccines but did not intend to receive the next seasonal influenza vaccine. To explore the possible reasons, we summarized the differences in the respondents’ characteristics in Table S3. It was of interest to find that respondents ages 11–18 had the highest percentage of those deciding not to get seasonal influenza vaccination. A possible reason might be that this age group was not a routine priority group for the seasonal influenza vaccination. One study in the United States also found that vaccination rate decreased with age, with high-school students having the lowest vaccination rate and elementary schoolchildren having the highest rate [34]. Perceiving good health status and frequently visiting public places were two factors linked to the intention not to get the next seasonal influenza vaccination. However, 87.0% of respondents with both pandemic (H1N1) vaccination and previous seasonal influenza vaccination experience intended to get the next seasonal influenza vaccination.

People with more contacts were more likely to get pandemic (H1N1) 2009 vaccines. A direct explanation for this phenomenon was not found in previous literature. Indirect evidence from Europe, however, indicated that vaccination target populations, such as the elderly, healthcare workers, and people suffering with chronic illness, would like to get seasonal influenza vaccines because they do not want to infect their friends and family [35]. Similar observations were also found in our results (Table 4). If the number of household members was equal to or larger than 5, the household included a member with a medical background, or someone in the household was under age 12 or over age 65, individuals had higher intention to receive seasonal influenza vaccines.

One interesting finding from this study was the negative effect of the habit of watching political talk shows on either receipt of the pandemic (H1N1) 2009 vaccination or intention to receive a seasonal influenza vaccine. During the pandemic (H1N1) 2009 vaccination in November 2009, some negative discussions on the safety of the vaccine were featured on TV political talk shows every day. That might partially explain why those respondents were less likely to get a pandemic (H1N1) 2009 vaccine or had a lower intention to receive seasonal influenza vaccine. One study in Canada also found that having negative beliefs about the pandemic (H1N1) 2009 vaccine and deciding not to be vaccinated were highly correlated [36].

There were some limitations in this study. The study was initiated right after the 2009/10 flu season. The acceptance of pandemic (H1N1) 2009 vaccinations might not be the same as the acceptance of seasonal influenza vaccine, due to differing perceptions of disease risk. The pandemic (H1N1) 2009 vaccination rate approximated the official coverage rate among the whole population. Therefore, the representativeness of the data could be assured. In Taiwan, most government-funded influenza vaccines were targeted on the priority populations. Therefore, some socio-demographic factors, such as age or working status, were also highly related to the specific population. In future studies, understanding the factors affecting healthy adults’ decisions to get influenza vaccinations at their own expense would be beneficial for health education.

## Conclusions

To our knowledge, this was the first study to compare the factors of pandemic (H1N1) 2009 vaccination and intentions to receive seasonal influenza vaccine at the same time. Since children, the elderly, and those with previous vaccination experiences are more likely both to have received pandemic vaccines and to intend to receive seasonal influenza vaccines, the school-based vaccination program and government-funded vaccines for the priority groups play crucial roles for promoting influenza vaccination in Taiwan. Successful vaccination campaigns during annual influenza seasons will be strong support for promoting the acceptance and delivery of novel influenza vaccines during pandemic periods. Perceptions of pandemic or worries about friends or family being infected are related to people’s intention to receive influenza vaccinations. Thus, prompt and clear risk communication about an influenza epidemic or pandemic through mass media can help generate the correct perception of the disease among the public and enhance acceptance of the vaccine.

## Supporting Information

Table S1 **Summary of variables and response categories.**
(DOCX)

Table S2 **The four ordinal variables associated with receipt of pandemic (H1N1) 2009 vaccination in** **Table 2** **.**
(DOCX)

Table S3 **Differences in characteristics of the respondents who received influenza vaccination but decided not to get the next seasonal influenza vaccination.** (A) Respondents who got pandemic (H1N1) influenza vaccination but decided not to get the next seasonal influenza vaccination (N = 548). (B) Respondents who got at least one seasonal influenza vaccination in the past five years but did not intend to get the next seasonal influenza vaccination (N = 551).
(DOCX)
