# Supplementary material for: Determinants of Receiving the Pandemic (H1N1) 2009 Vaccine and Intention to Receive the Seasonal Influenza Vaccine in Taiwan
Source: PLoS One. 2014 Jun 27;9(6):e101083. doi: 10.1371/journal.pone.0101083 (PMC4074160; doi:10.1371/journal.pone.0101083)
Supplement: Table S1 — Summary of variables and response categories. (DOCX) [file pone.0101083.s001.docx]

Table S1 Summary of variables and response categories

| Variables | Response categories |
| --- | --- |
| Gender | Males, Females |
| Age groups | Reclassified as: 0-18, 19-35, 36-50, 51-64, and 65 & older in Tables 2 & 3; 0-18 is further divided into 0-10 and 11-18 in Tables 4 & 5. |
| Education | Elementary, High school, College or higher |
| Working status | Pre-school, Students, Work, Unemployed |
| Self-reported health status | 1: Poor, 2: Fair, 3: Good, 4: Very good, 5: Excellent |
| Frequency of visiting public places | 1: Almost never, 2: Several times a year, 3: At least once a month, 4: At least once a week, 5: Almost every day |
| Habit of watching political talk shows | 0: Never watched, 1: Have watched |
| Perception of severity of pandemic in 2009 | 1:Not at all serious, 2: Not too serious, 3: Somewhat serious, 4: Very serious |
| Level of worry about a new pandemic | 1: Not at all worried, 2: Not too worried, 3: Somewhat worried, 4: Very worried |
| Previous vaccination against seasonal influenza | 0: Never, 1: At least one influenza vaccination within past five years |
| Household |  |
| # members>=5 | 0: 1-4 members, 1: >=5 members (median=4) |
| with med. background | 0: No, 1: Yes |
| someone under age 12 | 0: No, 1: Yes |
| someone over age 65 | 0: No, 1: Yes |
| Contact diary |  |
| # people>=10 | 0: 1-9 persons, 1: 10-40 persons (median=9) |
| % bodily contact | 0-1 (Contacts with bodily contact / All Contacts) |
