# Supplementary material for: Determinants of Receiving the Pandemic (H1N1) 2009 Vaccine and Intention to Receive the Seasonal Influenza Vaccine in Taiwan
Source: PLoS One. 2014 Jun 27;9(6):e101083. doi: 10.1371/journal.pone.0101083 (PMC4074160; doi:10.1371/journal.pone.0101083)
Supplement: Table S2 — The four ordinal variables associated with receipt of pandemic (H1N1) 2009 vaccination in Table 2 . (DOCX) [file pone.0101083.s002.docx]

Table S2 The four ordinal variables associated with receipt of pandemic (H1N1) 2009 vaccination in Table 2

| Variables | Received Vaccination (%) | |
| --- | --- | --- |
|  | No (n=1,406) | Yes (n=548) |
| Self-reported health status |  |  |
| 1: Poor | 7.3 | 6.2 |
| 2: Fair | 42.0 | 34.3 |
| 3: Good | 22.3 | 21.2 |
| 4: Very good | 21.5 | 27.0 |
| 5: Excellent | 6.9 | 11.3 |
| Frequency of visiting public places |  |  |
| 1: Almost never | 5.3 | 4.6 |
| 2: Several times a year | 10.0 | 5.7 |
| 3: At least once a month | 20.0 | 13.7 |
| 4: At least once a week | 31.9 | 16.4 |
| 5: Almost every day | 32.9 | 59.7 |
| Perception of severity of pandemic in 2009 |  |  |
| 1: Not at all serious | 3.2 | 2.3 |
| 2: Not too serious | 40.2 | 32.1 |
| 3: Somewhat serious | 39.1 | 40.2 |
| 4: Very serious | 17.6 | 25.4 |
| Level of worry about a new pandemic |  |  |
| 1:Not at all worried | 8.5 | 6.3 |
| 2:Not too worried | 29.0 | 27.4 |
| 3:Somewhat worried | 43.5 | 43.8 |
| 4:Very worried | 19.0 | 22.5 |
