# Supplementary material for: Determinants of Receiving the Pandemic (H1N1) 2009 Vaccine and Intention to Receive the Seasonal Influenza Vaccine in Taiwan
Source: PLoS One. 2014 Jun 27;9(6):e101083. doi: 10.1371/journal.pone.0101083 (PMC4074160; doi:10.1371/journal.pone.0101083)
Supplement: Table S3 — Differences in characteristics of the respondents who received influenza vaccination but decided not to get the next seasonal influenza vaccination. (A) Respondents who got pandemic (H1N1) influenza vaccination but decided not to get the next seasonal influenza vaccination (N = 548). (B) Respondents who got at least one seasonal influenza vaccination in the past five years but did not intend to get the next seasonal influenza vaccination (N = 551). (DOCX) [file pone.0101083.s003.docx]

Table S3 Differences in characteristics of the respondents who received influenza vaccination but decided not to get the next seasonal influenza vaccination

1. Respondents who got pandemic (H1N1) influenza vaccination but decided not to get the next seasonal influenza vaccination (N=548)

| Variables | Do not intend to  get vaccine | | Intend to get vaccine | | *p*-value |
| --- | --- | --- | --- | --- | --- |
|  | *n=278* | | *n=270* | |  |
| Gender, no.(%) |  |  |  |  | 0.271 |
| Male | 150 | (54.0) | 133 | (49.3) |  |
| Female | 128 | (46.0) | 137 | (50.7) |  |
| Age groups, no. (%) |  |  |  |  | <0.001 |
| 0 – 10 | 66 | (23.8) | 71 | (26.3) |  |
| 11 – 18 | 126 | (45.3) | 55 | (20.3) |  |
| 19 – 64 | 69 | (24.8) | 72 | (26.7) |  |
| 65 & older | 17 | (6.1) | 72 | (26.7) |  |
| Education, no. (%) |  |  |  |  | <0.001 |
| Elementary | 103 | (37.0) | 154 | (57.0) |  |
| High school | 135 | (48.6) | 75 | (27.8) |  |
| College or higher | 40 | (14.4) | 41 | (15.2) |  |
| Working status, no. (%) |  |  |  |  | <0.001 |
| Pre-school | 19 | (6.8) | 30 | (11.1) |  |
| Students | 174 | (62.6) | 95 | (35.2) |  |
| Work | 54 | (19.4) | 69 | (25.6) |  |
| Unemployed | 31 | (11.2) | 76 | (28.1) |  |
| Self-reported health status, median (IQR) | 3 | (2-4) | 3 | (2-4) | <0.001 |
| Frequency of visiting public places, median (IQR) | 5 | (4-5) | 5 | (3-5) | <0.001 |
| Habit of watching political talk shows, no. (%) | 47 | (16.9) | 65 | (24.1) | 0.038 |
| Perception of severity of pandemic in 2009, median (IQR) | 3 | (2-4) | 3 | (2-3.5) | 0.142 |
| Level of worry about a new pandemic, median (IQR) | 3 | (2-3) | 3 | (2-3.5) | 0.072 |
| Previous vaccination against seasonal influenza, no. (%) | 96 | (34.5) | 224 | (87.0) | <0.001 |
| Household, no. (%) |  |  |  |  |  |
| # members>=5 | 130 | (46.8) | 127 | (47.0) | 0.949 |
| with med. background | 20 | (7.2) | 32 | (11.9) | 0.063 |
| someone under age 12 | 87 | (31.3) | 104 | (38.5) | 0.076 |
| someone over age 65 | 77 | (27.7) | 92 | (34.1) | 0.106 |
| Contact diary |  |  |  |  |  |
| # people>=10, no. (%) | 168 | (60.9) | 154 | (57.3) | 0.390 |
| % bodily contact, median (IQR) | 0.40 | (0.2-0.6) | 0.35 | (0.2-0.7) | 0.901 |

IQR*interquartile range* (25th percentile-75th percentile)

1. Respondents who got at least one seasonal influenza vaccination in the past five years but did not intend to get the next seasonal influenza vaccination (N=551)

| Variables | Do not intend to  get vaccine | | Intend to get vaccine | | *p*-value |
| --- | --- | --- | --- | --- | --- |
|  | *n=194* | | *n=357* | |  |
| Gender, no.(%) |  |  |  |  | 0.951 |
| Male | 94 | (48.5) | 172 | (48.2) |  |
| Female | 100 | (51.5) | 185 | (51.8) |  |
| Age groups, no. (%) |  |  |  |  | <0.001 |
| 0 – 10 | 46 | (23.7) | 86 | (24.1) |  |
| 11 – 18 | 48 | (24.7) | 46 | (12.9) |  |
| 19 – 64 | 64 | (33.0) | 95 | (26.6) |  |
| 65 & older | 36 | (18.6) | 130 | (36.4) |  |
| Education, no. (%) |  |  |  |  | 0.001 |
| Elementary | 89 | (45.9) | 223 | (62.5) |  |
| High school | 62 | (31.9) | 81 | (22.7) |  |
| College or higher | 43 | (22.2) | 53 | (14.8) |  |
| Working status, no. (%) |  |  |  |  | <0.001 |
| Pre-school | 21 | (10.8) | 43 | (12.9) |  |
| Students | 80 | (41.3) | 87 | (24.4) |  |
| Work | 52 | (26.8) | 97 | (27.2) |  |
| Unemployed | 41 | (21.1) | 127 | (35.5) |  |
| Self-reported health status, median (IQR) | 3 | (2-4) | 3 | (2-4) | 0.008 |
| Frequency of visiting public places, median (IQR) | 5 | (3-5) | 4 | (3-5) | 0.014 |
| Habit of watching political talk shows, no. (%) | 48 | (24.7) | 90 | (25.2) | 0.904 |
| Perception of severity of pandemic in 2009, median (IQR) | 3 | (2-4) | 3 | (2-3) | 0.082 |
| Level of worry about a new pandemic, median (IQR) | 3 | (2-3) | 3 | (2-3) | 0.081 |
| Household, no. (%) |  |  |  |  |  |
| # members>=5 | 84 | (43.3) | 165 | (46.2) | 0.511 |
| with med. background | 31 | (16.0) | 48 | (13.5) | 0.425 |
| someone under age 12 | 68 | (35.1) | 133 | (37.3) | 0.608 |
| someone over age 65 | 61 | (31.4) | 134 | (37.5) | 0.153 |
| Contact diary |  |  |  |  |  |
| # people>=10, no. (%) | 106 | (55.2) | 190 | (53.5) | 0.705 |
| % bodily contact, median (IQR) | 0.38 | (0.1-0.6) | 0.33 | (0.1-0.7) | 0.907 |

IQR*interquartile range* (25th percentile-75th percentile)
